# Identifying optimal parameters for infrared neural stimulation in the peripheral nervous system

**DOI:** 10.1117/1.NPh.8.1.015012

**Published:** 2021-03-31

**Authors:** Graham Throckmorton, Jonathan Cayce, Zane Ricks, Wilson R. Adams, Eric Duco Jansen, Anita Mahadevan-Jansen

**Affiliations:** a Vanderbilt Biophotonics Center, Keck FEL Center, Nashville, Tennessee, United States; b Vanderbilt University, Department of Biomedical Engineering, Nashville, Tennessee, United States; c Vanderbilt University Medical Center, Department of Neurological Surgery, Nashville, Tennessee, United States

**Keywords:** infrared neural stimulation, efficacy, clinical translation, peripheral nerves, *in vivo*, neurophotonics

## Abstract

**Significance:** Infrared neural stimulation (INS) utilizes pulsed infrared light to selectively elicit neural activity without exogenous compounds. Despite its versatility in a broad range of biomedical applications, no comprehensive comparison of factors pertaining to the efficacy and safety of INS such as wavelength, radiant exposure, and optical spot size exists in the literature.

**Aim:** Here, we evaluate these parameters using three of the wavelengths commonly used for INS, 1450 nm, 1875 nm, and 2120 nm.

**Approach:** In an *in vivo* rat sciatic nerve preparation, the stimulation threshold and transition rate to 100% activation probability were used to compare the effects of each parameter.

**Results:** The pulsed diode lasers at 1450 nm and 1875 nm had a consistently higher ($∼1.0\;J/{\mathrm{cm}}^{2}$) stimulation threshold than that of the Ho:YAG laser at 2120 nm ($∼0.7\;J/{\mathrm{cm}}^{2}$). In addition, the Ho:YAG produced a faster transition rate to 100% activation probability compared to the diode lasers. Our data suggest that the superior performance of the Ho:YAG is a result of the high-intensity microsecond spike at the onset of the pulse. Acute histological evaluation of diode irradiated nerves revealed a safe range of radiant exposures for stimulation.

**Conclusion:** Together, our results identify measures to improve the safety, efficacy, and accessibility of INS technology for research and clinical applications.

## 1 Introduction

For the last century, scientists and clinicians have used the direct application of electrical current to modulate neural activity and probe the structure and function of the nervous system.^1^^–^^4^ To this day, electrical stimulation remains the gold standard for assessing and quantifying neural activity. Despite its broad utility, electrical stimulation is hindered by fundamental limitations, namely, current spread and stimulation artifacts. Current spread, in which the injected current disperses into the surrounding tissue, restricts the spatial specificity of neural activation.^5^^,^^6^ Spatial specificity is both clinically and experimentally advantageous when targeting specific regions of a nerve or other neural structures such as the brain and cochlea which have a high degree of spatial dependence.^7^^–^^9^ Clinically, current spread in deep brain stimulation cases can cause deleterious side effects that may hinder the effective treatment of Parkinson’s disease.^10^^–^^12^ In the peripheral nervous system, current spread can directly induce muscle contractions that may falsely indicate nerve viability in damaged nerve segments.^13^^–^^15^ Improving the spatial specificity and overcoming current spread requires precise control over electrode placement and/or necessitates direct electrical coupling to the neuron, as done in patch-clamp techniques. In addition to current spread, electrical stimulation artifacts impede the ability to record proximal to the stimulation site because the artifact can mask the true electrophysiological signal.^6^ Stimulation artifacts can also impair the interpretation of nerve graft viability and of electrophysiological recordings during intraoperative nerve monitoring.^5^^,^^16^^,^^17^ Thus, neural stimulation techniques providing spatially confined excitation without stimulation artifacts represent highly desired tools in both clinical and experimental settings.

Initial studies published on infrared neural stimulation (INS),^5^^,^^18^^–^^20^ along with the foundational work in optogenetics,^21^ demonstrated the ability to optically stimulate selective neuronal populations without stimulation artifacts or tissue contact. While optogenetics relies on the genetic modification of the neural tissue to express light-sensitive ion channels, INS intrinsically elicits neural through tissue absorption of short-wave infrared (SWIR) light.^18^ Moreover, the inherent spatial precision of INS is a direct result of the rapid spatiotemporal thermal gradient INS creates.^18^ The thermal energy from the infrared pulses is spatially confined to the irradiated volume as determined by the laser spot size and the penetration depth of the light in tissue. The work of Shapiro et al. further shows how the deposition of thermal energy generates an action potential.^22^

During INS, the transient heating induced by water absorption causes a rapid change in cell membrane capacitance leading to the initiation of an action potential.^22^ In a subsequent study, Plaksin et al. showed that a thermal-mechanical effect causes the membrane to thin axially and expand laterally producing the change in cell membrane capacitance.^23^ Though researchers still debate the exact mechanism, this capacitive change appears to evoke action potentials through a universal mechanism triggered by thermal-mechanical effects within the extracellular phospholipid bilayer.^22^^–^^28^ Despite the incomplete understanding of the biophysical mechanism of INS, the innate label-free nature and high degree of spatial specificity of this technique make INS well suited for clinical translation.

Previously, the feasibility of INS in humans was demonstrated by activating specific functional connections in human dorsal rootlets more precisely than electrical stimulation.^29^ In the central nervous system, researchers have used INS for cortical mapping of the non-human primate visual system and modulating task-specific behavior.^8^^,^^9^^,^^30^^–^^32^ The application of INS in animal models for surgical guidance includes use in the prostate, bladder, and base of the skull to minimize collateral nerve damage that can give rise to urinary incontinence, sexual impotence, and facial paralysis, respectively.^16^^,^^17^^,^^33^^–^^35^ Together, these studies substantiate INS as a potentially valuable clinical tool in both the central and peripheral nervous systems.

Despite its proven capability as a clinical tool, the cost and size of lasers systems used for INS present a major barrier to its widespread use. Initially, INS studies used bulky and expensive flashlamp-pumped solid state lasers. As the field progressed, cheaper diode laser systems became more widely adopted in laboratories utilizing INS. Using these lower-cost diode systems (∼$20k), multiple groups published results demonstrating the efficacy of 1875 nm and 1450 nm light for eliciting neural activity.^9^^,^^22^^,^^26^^,^^27^^,^^30^^–^^32^^,^^36^^–^^51^ Custom diode systems can be made for a few thousand dollars but require some proficiency in laser hardware and electronics. Other groups opted to use fiber lasers with tunable thulium fiber lasers centered around 2000 nm being the most common, but this wavelength may be too strongly absorbed by water in the tissue.^52^^–^^56^ Many groups chose to construct custom laser systems to study INS, which largely accounts for the variability in parameters used throughout the literature. To make INS an accessible and reliable tool for clinical neural modulation, a need exists to evaluate the optimal laser parameters that result in safe and effective stimulation, including pulse duration, spot size, and radiant exposure. In addition, despite the multitude of studies performed using 1450 nm light, no histological evaluation of the safety for INS at 1450 nm exists. However, no histological or functional damage was observed in the acute studies using the Ho:YAG laser at 2120 nm or diode lasers at 1875 nm.^29^^,^^31^^,^^57^^,^^58^ A safety ratio (the ratio of the ablation threshold to stimulation threshold) of 2:1 was established.^29^^,^^57^ Due to the thermal gradient inherent to INS, histological safety represents a crucial consideration for future experimental and translational efforts.^29^^,^^31^^,^^56^^,^^57^

The goal of this study is to identify optimal stimulation parameters across three SWIR wavelength lasers, two diode lasers centered at 1450 nm and 1875 nm, and the “gold standard” Ho:YAG laser at 2120 nm, to empirically evaluate the efficacy and histological safety of these wavelengths in an *in vivo* rat sciatic nerve model. The effect of spot size, pulse width, and radiant exposure on the efficacy and safety of INS was investigated. Our results identify strategies to maximize the efficacy and safety of diode lasers for INS while improving the accessibility and utility of INS for future research and clinical applications.

## 2 Materials and Methods

All experiments were conducted at the Vanderbilt Biophotonics Center in adherence to protocols approved by the Vanderbilt Institution of Animal Care and Use Committee.

### 2.1 Animal Preparation

**Fig. 1:**
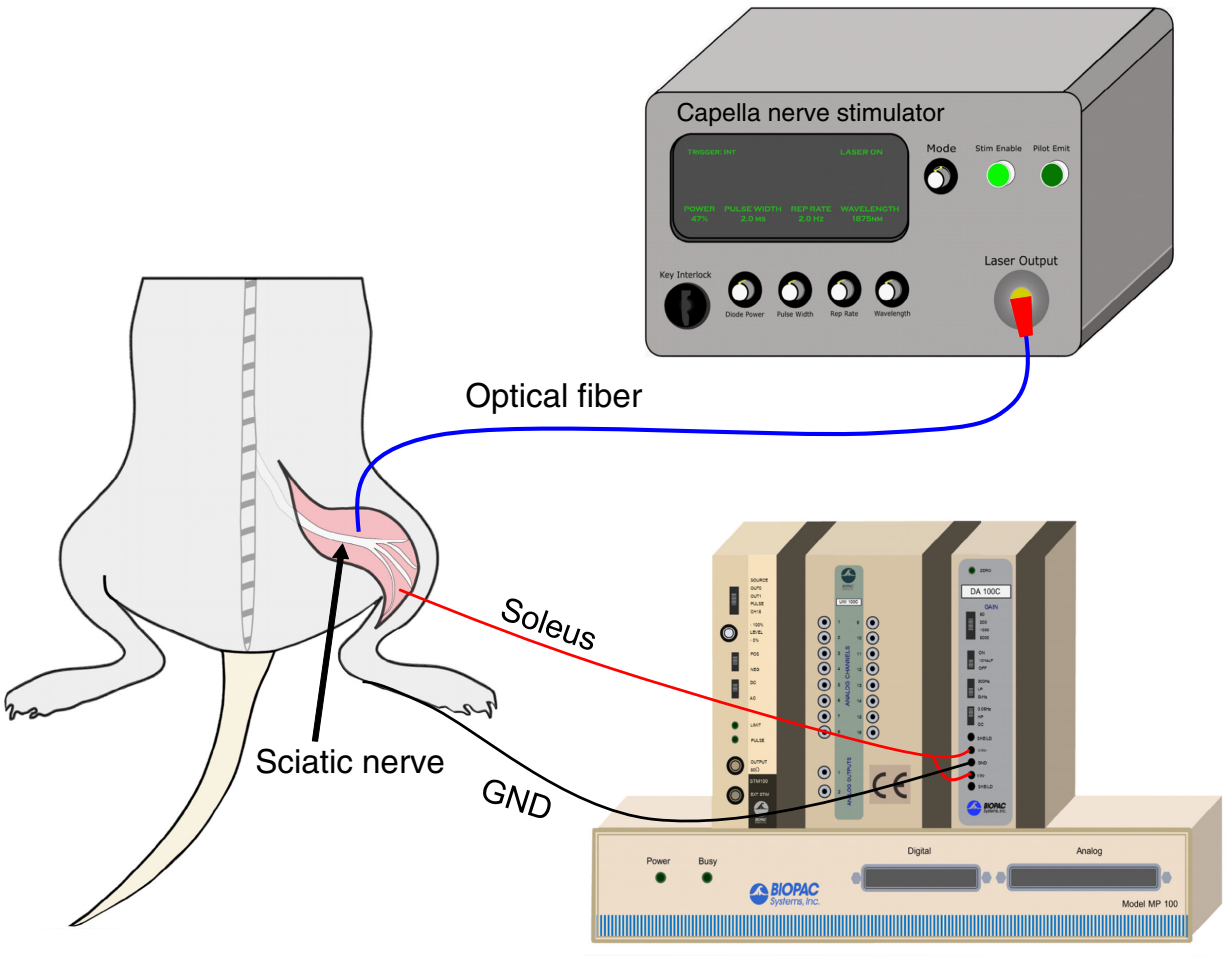
Experimental setup for *in vivo* INS of the rat sciatic nerve. The laser source was coupled directly into a multimode optical fiber positioned over the nerve. The nerve was stimulated optically and CMAPs were recorded from the soleus muscle using a modular data acquisition system.

*In vivo* sciatic nerve experiments were performed using adult male Sprague-Dawley rats (n=26, 300 to 350 g) as a mammalian model. Animals were anesthetized by inhalation of isoflurane (3%, 3  L/min) and maintained under sedation (2% to 2.5%, 1.5  L/min) for the duration of the experiments. Once anesthetized, animals were placed on a polycarbonate platform and fitted with a nose cone to maintain anesthesia. A water-circulating heating pad (catalog# 40-90-8, FHC, Bowdoin, Maine) was used to maintain a body temperature of 36°C to 37°C throughout experiments while the animal’s body temperature was monitored using a rectal probe. Both hind limbs were shaved, and the dorsal surface of the feet were secured to the edge of the platform. A 3-cm incision was made posterior-laterally extending from the gluteus muscles to the popliteal region. The skin was separated from the underlying tissue to expose the biceps femoris, which was carefully incised to expose the sciatic nerve (Fig. 1). The muscle fascia overlying the surface of the nerve was delicately removed to expose the nerve surface. The epineurium was left intact. Nerves were periodically bathed in room temperature sterile saline throughout experiments to maintain tissue hydration and prevent desiccation. After the experiment, each rat was euthanized via anesthetic overdose followed by cervical dislocation that adhered to the IACUC approved protocol.

### 2.2 Electrophysiology

Compound muscle action potentials (CMAPs) were recorded from paired, bipolar subdermal needle electrodes (Medtronic Xomed, Jacksonville, Florida) inserted into the soleus. A subdermal grounding electrode was also inserted into the foot of the same leg. CMAPs and the trigger output of the laser were recorded simultaneously with a modular data acquisition system (MP100, Biopac Systems Inc., Santa Barbara, California) using Acknowledge^®^ software (Biopac Systems Inc., Santa Barbara, California). CMAPs were sampled at 6500 Hz and captured digitally after amplification using a differential amplifier (DA 100C, Biopac Systems Inc., Santa Barbara, California). All signals were amplified 1000× and bandpass filtered from 0.05 to 5000 Hz. Before each experiment, the viability of the sciatic nerve was assessed with electrical stimulation (0.3 to 0.4 V at 500  μs) using a Prass standard monopolar stimulator probe (Medtronic Xomed, Jacksonville, Florida). Only CMAPs occurring within 5 ms of the infrared pulse were included in data analysis. Representative CMAP traces can be found in Fig. S1 in the Supplemental Materials.

### 2.3 Infrared Neural Stimulation

A parametric assessment of stimulation thresholds was performed with a total of four SWIR wavelengths and three spot sizes across a range of pulse widths and radiant exposures (Table 1). The lasers used for these experiments included two laser diode systems at 1450 nm and 1875 nm, respectively (Capella, Lockheed Martin-Aculight, Bothell, Washington), a custom laser diode system at 1470 nm (Innovative Photonics Solutions, Monmouth Junction, New Jersey), and a Ho:YAG laser at 2120 nm (Model 1-2-3 laser, Schwartz Electro Optics, Inc., Concord, Massachusetts).

**Table 1:** INS parameters.

Laser	Wavelength (nm)	Water absorption coefficient^a^ (${\mathrm{cm}}^{-1}$)	Pulse width (μs)	Spot size (μm)	Radiant exposures ($J/{\mathrm{cm}}^{2}$)
Diode	1450	29.7	2000	500 to 1000	0 to 2.5
	1470	24.9	350	500	0 to 2.5
	1875	25.4	2000, 3000, 5000	500 to 1000	0 to 2.5
Ho:YAG	2120	24	350^b^	500 to 1000	0 to 1.0

a Water absorption coefficients are based on the cubic interpolation of data acquired by Hale and Querry (1973).^59^

b Full width half maximum (FWHM).

#### 2.3.1 Radiant exposure and pulse duration

For all diode laser experiments at a given spot size, the diode current was adjusted to vary the radiant exposure between 0 and $2.5\;J/{\mathrm{cm}}^{2}$. In experiments with the 1875 nm diode laser, the pulse duration was modulated for a given spot size to produce the necessary radiant exposures (e.g., 2 ms pulses for 500  μm spot size and 5 ms pulses for 1000  μm). For the Ho:YAG laser, the radiant exposures were modulated with attenuators placed in the beam path. For each experimental trial, nerves were irradiated with a 10-s pulse train at 2 Hz for a total of 20 pulses at a randomized radiant exposure.

Due to the limited power output of the 1450 nm and 1875 nm diode lasers, the 1470 nm diode laser was used to generate 350  μs pulses with equal energy to that of the Ho:YAG laser. The 1470 nm diode laser was only used to examine the effects of the pulse width. All pulse widths refer to the full width at half maximum of the pulse.

#### 2.3.2 Spot size

**Fig. 2:**
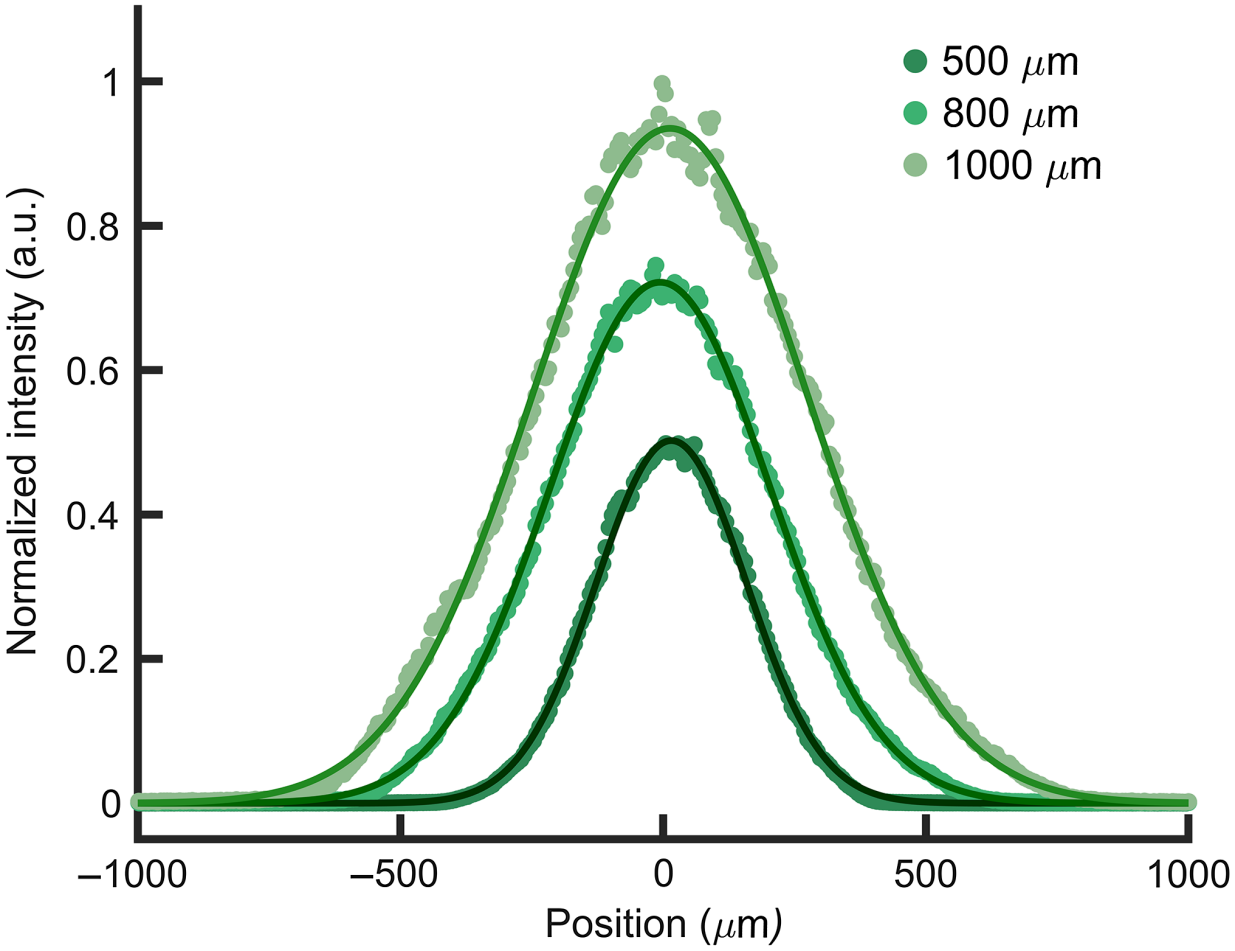
Spot size measurements made using IR beam profiler fitted to a Gaussian distribution (solid lines). Maximum intensities are modulated for clear visualization. ${S\text{pot Size}}_{500}=528.5\;\mu m$, $R^{2}=0.9989$; ${S\text{pot Size}}_{800}=802.1\;\mu m$, $R^{2}=0.9993$; ${S\text{pot Size}}_{1000}=1003\;\mu m$, $R^{2}=0.9986$.

Stimulation thresholds were determined for three spot sizes: 500  μm (504.5±20  μm), 800  μm (805.6±18.8  μm), and 1000  μm (1019±29.8  μm) (Fig. 2). All spot sizes were measured using an infrared beam profiler (BP209-IR2, Thorlabs, Newton, New Jersey), validated using the knife-edge technique,^60^ and refer to the $1/e^{2}$ diameter of the beam profile. Spot sizes were modulated by adjusting the distance between the bare 400  μm diameter optical fiber (NA=0.22; Ocean Optics, Dunedin, Florida) output and the nerve surface. For the 500, 800, and 1000  μm spot sizes, the average distance away from the nerve surface was 862.9±21.79, 1874±156, and 2646±240  μm, respectively. The fiber probes were positioned using a micromanipulator (World Precision Instruments, Sarasota, Florida) orthogonal to the main trunk of the sciatic nerve.

#### 2.3.3 Spikeless Ho:YAG pulse

**Fig. 3:**
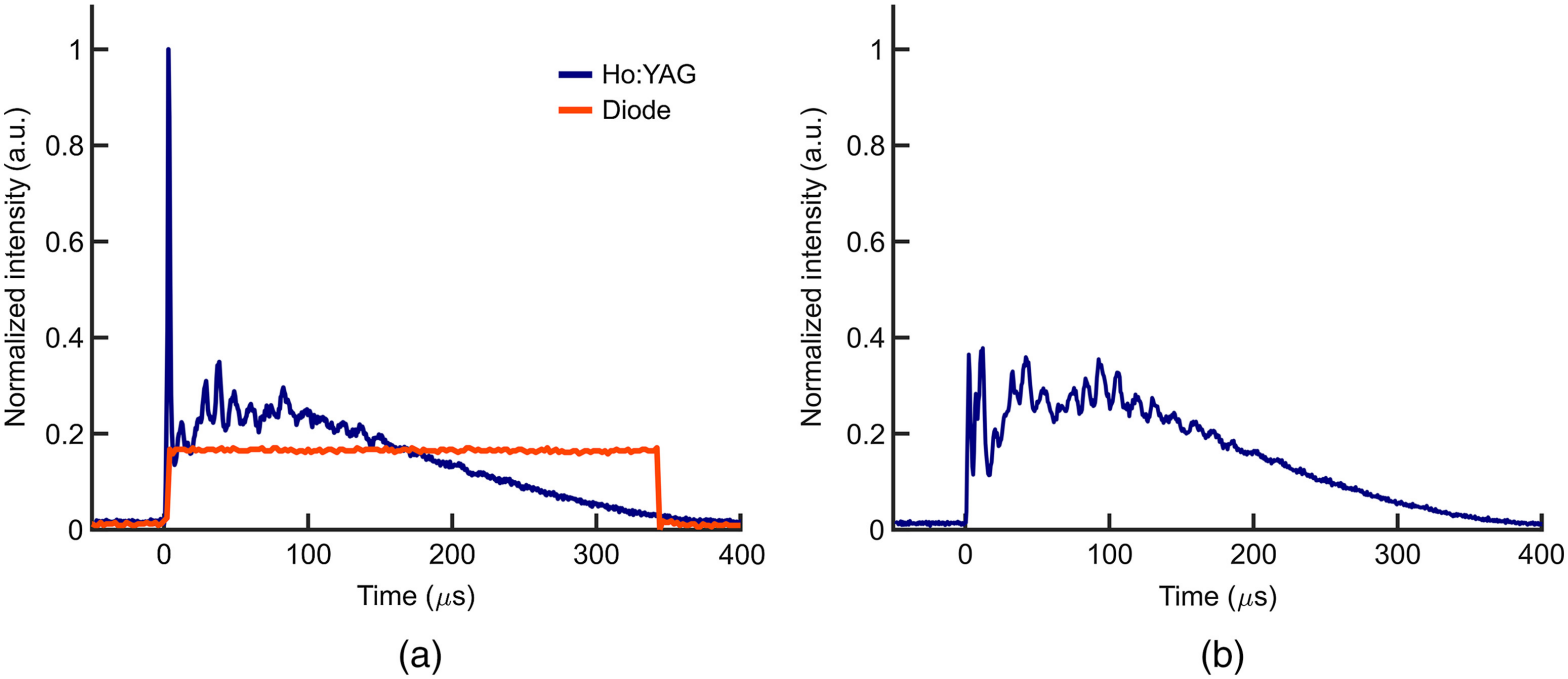
AUC-normalized temporal pulse shapes from Ho:YAG and diode lasers. (a) AUC-normalized temporal pulse shapes from a regular Ho:YAG and 350  μs diode pulse. (b) AUC-normalized temporal pulse shape from a spikeless Ho:YAG pulse. Pulse traces were taken with amplified, InGaAs detector (PDA10D, Thorlabs, Newton, New Jersey).

To investigate the effects of the leading spike in the Ho:YAG pulse on INS efficacy [Fig. 3(a)], the spike was eliminated using a 200-μm-thick glass chamber filled with deionized water placed just outside the laser chamber in the beam path. To achieve spikeless pulses, the pulse energy of the Ho:YAG was increased until the initial microsecond spike at the beginning of the pulse contained the necessary energy to create a vapor bubble.^61^ Once the bubble was generated from the spike’s energy, the spikeless remainder of the Ho:YAG pulses was able to pass through the vapor with minimal absorption and be coupled into the fiber [Fig. 3(b)]. Temporal pulse shapes were continually monitored using an InGas amplified photodetector (PDA10D2, Thorlabs, Newton, New Jersey) connected to an oscilloscope (Tekronix, Beaverton, Oregon). If the spike was not eliminated for a pulse, that pulse was not used for data analysis, and the number of pulses in the train was increased until 20 spikeless pulses were delivered to the nerve.

### 2.4 Data Analysis

The stimulation threshold ($H_{50}$) is defined as the radiant exposure at which 50% of the laser pulses evoked CMAP responses, and this definition is used to compare all data. To determine the stimulation threshold, recordings from each trial were used to determine the number of INS-evoked CMAPs. It is important to note that CMAP amplitude increases with the magnitude of radiant exposure.^20^^,^^62^ For the purposes of this study, the presence of a CMAP regardless of its amplitude is used as the physiological end point to indicate successful INS, and thus the probability of evoking a CMAP is unaffected. Responses with a peak magnitude greater than two standard deviations above the baseline were considered evoked CMAPs. The number of evoked CMAPs was then divided by the total number of delivered pulses to determine the activation probability for every radiant exposure. A cumulative distribution function (CDF) of the standard normal distribution

$$F(x;\mu ,\sigma^{2})=\frac{1}{2}[1+\mathrm{erf}(\frac{x-\mu }{\sigma \sqrt{2}})],\;x\in R,$$ (1)

where x is the radiant exposure with mean μ and variance $\sigma^{2}$, was then fit to the data to determine the radiant exposure corresponding to μ or the 50% probability of evoking a CMAP ($H_{50}$).^63^ While the $H_{50}$ is of little use for practical applications, this method is a common means to determine and compare dosimetric thresholds for radiation bioeffects.^63^^–^^68^ INS parameters with a lower $H_{50}$ are considered more efficacious as they require less energy and produce smaller temperature rises.

Another measure of stimulation efficacy is the transition rate to 100% activation probability. The transition rate to 100% activation probability is defined as the peak slope of the fitted CDF ($m_{\text{peak}}$)

$$m_{\text{peak}}=\max[F^{\prime }(x;\mu ,\sigma^{2})],\;x\in R,$$ (2)

where $F^{\prime }$ denotes the first derivative of F. This calculation represents the determinacy of the stimulation threshold. A larger $m_{\text{peak}}$ (i.e., a sharper transition rate) translates to a more reliable and predictable stimulation. This characteristic is more desirable since a smaller range of radiant exposures exists where one may experience ambiguity as to whether a given pulse evokes an action potential or not. Graphical representations of $H_{50}$ and $m_{\text{peak}}$ calculations can be found in Fig. S2 in the Supplemental Materials.

### 2.5 Histological Evaluation

Twelve rats were prepared using the surgical protocol described previously to expose both sciatic nerves. Five different sites on each nerve were irradiated under five different experimental conditions using a bare polished fiber at a spot size of 500  μm: three experimental spots, a negative control spot, and a positive damage control spot. All five irradiation sites were spaced 1 mm apart and distributed axially along the nerve. All conditions were identical to the experimental protocol described above: a 10-s pulse train at a repetition rate of 2 Hz. The three experimental spots for each nerve were assessed at one, two, and three times the $H_{50}$. Radiant exposures of $∼6.0\;J/{\mathrm{cm}}^{2}$ served as the positive damage control. The negative control was applied by positioning the fiber similarly to other trials while omitting irradiation. These experimental conditions were evaluated for damage with the 1450 nm and 1875 nm laser systems. Each irradiation site was marked with histological tissue dye (FisherBrand, Leicestershire, United Kingdom) for excision and histological preparation. After stimulation, nerves were immediately excised and placed in 4% paraformaldehyde for 48 h. Fixed samples were then paraffin-embedded, sliced, and stained with toluidine blue to assess myelin damage. Histological slices were imaged under 60× magnification and examined for evidence of disruption and vacuolization of the myelin sheath, disruption of axons, ablation crater formation, and charring.^57^ Previous studies have rigorously determined the damage threshold for the Ho:YAG laser using the same criteria.^57^

### 2.6 Statistical Analysis

All statistical testing consisted of an ANOVA followed by a multiple comparison test using the Bonferroni method to account for small sample sizes.

## 3 Results

### 3.1 Spot Size

**Fig. 4:**
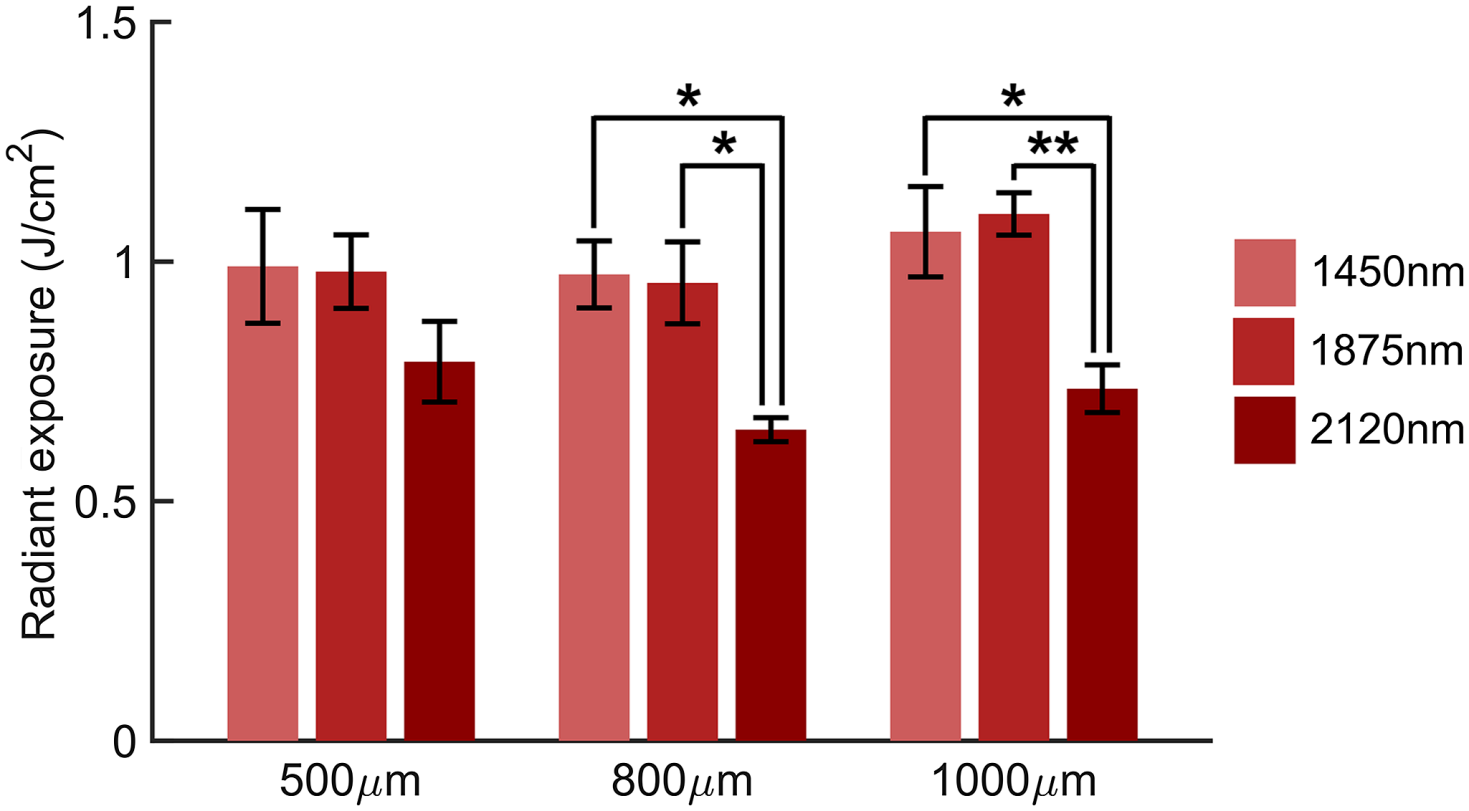
Stimulation threshold remains constant across every spot size for a given wavelength while the Ho:YAG consistently produces a $H_{50}$ lower than that of both laser diode systems. ($H_{50}\pm \mathrm{SEM}$, n=5).

For each of the wavelengths, the $H_{50}$ remained consistent across all spot sizes (Fig. 4). The laser diode systems at 1450 nm and 1875 nm have a combined average $H_{50}$ of $1.01\pm 0.06\;J/{\mathrm{cm}}^{2}$ while the average $H_{50}$ for the Ho:YAG is $0.73\pm 0.07\;J/{\mathrm{cm}}^{2}$ across all spot sizes. No substantial difference between the stimulation efficacy of 1450 nm and 1875 nm diode lasers was observed (p>0.49 for all comparisons). The $H_{50}$ for the Ho:YAG was lower than the diode lasers for every spot size. This difference was not significant (p=0.29) for the 500 μm spot size but was significant for the 800  μm (p<0.02 for 1450 nm and 1875 nm comparisons) and 1000  μm spot sizes (p<0.014 for 1450 nm and 1875 nm comparisons).

Note, diode lasers at 1875 nm tend to have less power output than their 1450 nm counterparts. Consequently, the radiant exposures at 500  μm, 800  μm, and 1000  μm spot sizes for the 1875 nm diode were achieved using 2 ms, 3 ms, and 5 ms pulses, respectively. Despite the change in pulse width, however, the $H_{50}$ for 1875 nm was unaltered for all spot sizes.

### 3.2 Pulse Width

**Fig. 5:**
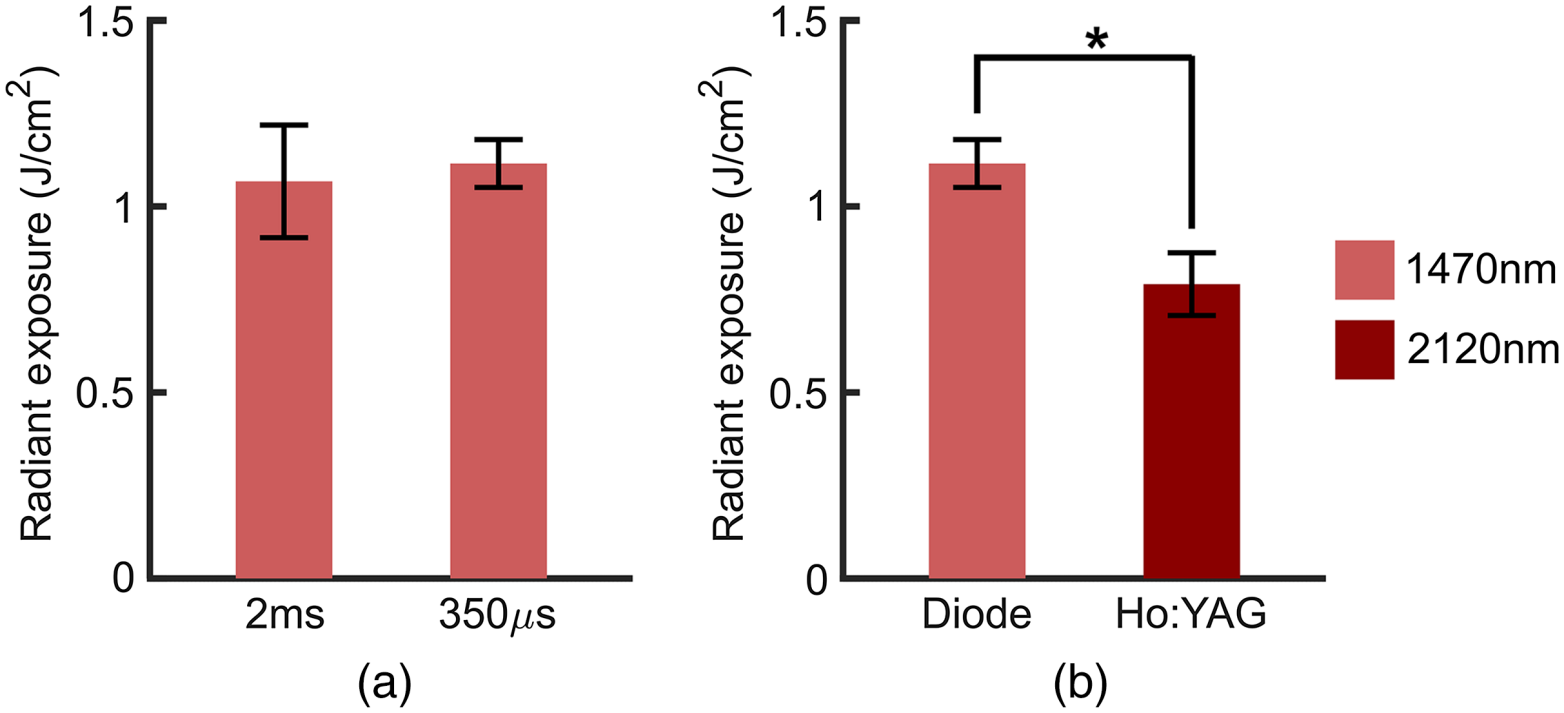
Effects of pulse width on $H_{50}$. (a) Pulse width does not significantly alter the $H_{50}$ for diode lasers ($H_{50}\pm \mathrm{SEM}$, n=5, 500  μm spot size). (b) $H_{50}$ of the Ho:YAG laser is lower than that of the diode lasers at equal pulse widths ($H_{50}\pm \mathrm{SEM}$, n=5, $\tau_{p}=350\;\mu s$, 500  μm spot size).

The effects of pulse width on $H_{50}$ were examined since the Ho:YAG produced a noticeably lower $H_{50}$ with 350  μs pulses compared to the ≥2  ms pulses from the diode lasers (Fig. 5). To compare pulse widths, a 1470 nm diode laser with the power specifications to produce adequate radiant exposures at 350  μs pulses was used to investigate the role of pulse duration on INS efficacy. In addition, the absorption coefficient of water at 1470 nm ($\mu_{a}\approx 24.9\;{\mathrm{cm}}^{-1}$) is also more similar to the water absorption coefficient at 2120 nm ($\mu_{a}\approx 24\;{\mathrm{cm}}^{-1}$) than at either 1450 nm or 1875 nm (Table 1). At a spot size of 500  μm, the $H_{50}$ did not change significantly between pulse widths of 2 ms and 350  μs [Fig. 5(a), p=0.38]. Further, shortening of the pulse width did not account for the reduced $H_{50}$ of the Ho:YAG [Fig. 5(b)]. Despite having equal spot sizes and pulse widths, the $H_{50}$ of the Ho:YAG remained significantly lower than that of the 1470 nm laser diode (p=0.016).

### 3.3 Transitions in Activation Probability of Diode versus Ho:YAG Lasers

**Fig. 6:**
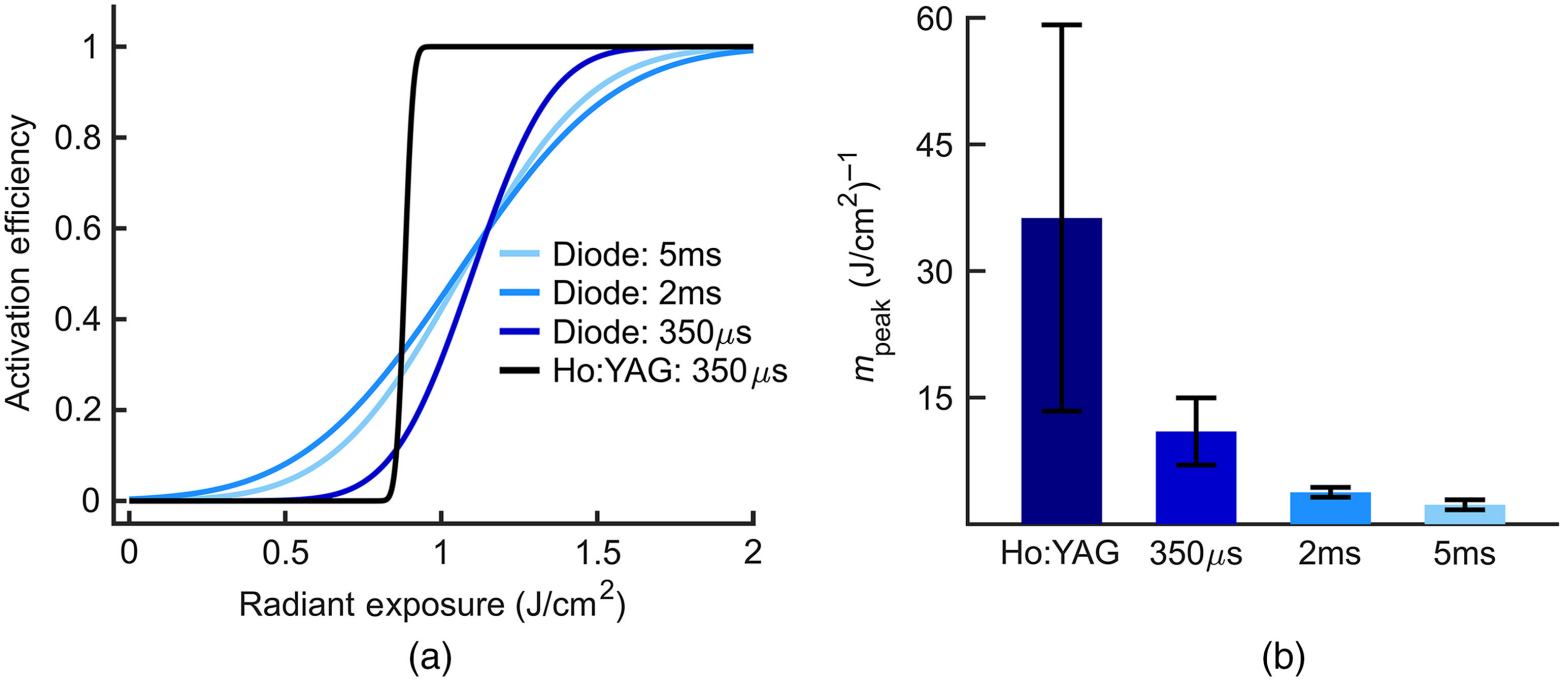
Difference in activation probabilities between diode and Ho:YAG lasers. (a) CDFs fitted to all samples from diode and Ho:YAG lasers for a given set of parameters. ($\lambda_{5\;\mathrm{ms}}=1875\;\mathrm{nm}$, ${\text{S}\text{pot Size}}_{5\;\mathrm{ms}}=1000\;\mu m$; $\lambda_{2\;\mathrm{ms}}=1470\;\mathrm{nm}$, ${\text{S}\text{pot Size}}_{2\;\mathrm{ms}}=500\;\mu m$; $\lambda_{350\;\mu s}=1470\;\mathrm{nm}$, $\lambda_{2\;\mathrm{ms}}=1470\;\mathrm{nm}$, ${\text{S}\text{pot Size}}_{350\;\mu s}=500\;\mu m$; $\lambda_{\mathrm{Ho}:\mathrm{YAG}}=2120\;\mathrm{nm}$, ${\text{S}\text{pot Size}}_{\mathrm{Ho}:\mathrm{YAG}}=500\;\mu m$). (b) The transition rate ($m_{\text{peak}}$) of diode lasers at various pulse widths and Ho:YAG laser at 350  μs pulse width ($m_{\text{peak}}\pm \mathrm{SEM}$, n=5). A larger $m_{\text{peak}}$ corresponds to a steeper transition in activation probability.

The change in activation probability resulting from the various laser systems was further evaluated by comparing the CDFs of activation probability (Fig. 6). Combined CDFs based on all data collected for a defined experimental condition are depicted in Fig. 6(a) ($\lambda_{5\;\mathrm{ms}}=1875\;\mathrm{nm}$, ${\text{S}\text{pot Size}}_{5\;\mathrm{ms}}=1000\;\mu m$; $\lambda_{2\;\mathrm{ms}}=1470\;\mathrm{nm}$, ${\text{S}\text{pot Size}}_{2\;\mathrm{ms}}=500\;\mu m$; $\lambda_{350\;\mu s}=1470\;\mathrm{nm}$, $\lambda_{2\;\mathrm{ms}}=1470\;\mathrm{nm}$, ${\text{S}\text{pot Size}}_{350\;\mu s}=500\;\mu m$; $\lambda_{\mathrm{Ho}:\mathrm{YAG}}=2120\;\mathrm{nm}$, ${\text{S}\text{pot Size}}_{\mathrm{Ho}:\mathrm{YAG}}=500\;\mu m$). Note that the 5 ms results are based on experiments using 1875 nm diode laser. Since pulse duration did not affect the $H_{50}$ as seen in Fig. 5, this is unlikely to affect the results presented here. The combined CDFs of the laser diode systems at 5 ms, 2 ms, and 350  μs remain relatively broad and gradually transition to an activation probability of 1. The distribution of the combined CDF for the Ho:YAG, however, is noticeably narrow with a steeper transition from an activation probability of 0 to 1. This steep transition is reflected in the $m_{\text{peak}}$ for each laser and pulse width.

The transition to an activation probability of 1 can be quantified by the peak slope of the CDF, $m_{\text{peak}}$. A larger $m_{\text{peak}}$ implies that there is a steeper transition from an activation probability of 0 to 1 that occurs quickly over a smaller range of radiant exposures than an activation CDF with a smaller $m_{\text{peak}}$. In comparison to the diode lasers, the $m_{\text{peak}}$ of the Ho:YAG is considerably higher [Fig. 6(b)]. The $m_{\text{peak}}$ of the diodes increases with decreasing pulse width while the $m_{\text{peak}}$ of the Ho:YAG is greater than that of the 1470 nm diode laser at the same pulse width.

### 3.4 Effects of Ho:YAG Pulse Shape on Stimulation Efficacy

In light of the lower $H_{50}$ and greater $m_{\text{peak}}$ of the Ho:YAG laser at equivalent pulse widths and radiant exposures to the diode lasers, the effect of the temporal pulse shape of the Ho:YAG was investigated. In comparing the pulse shapes of the two lasers, the most distinct feature of the asymmetric Ho:YAG pulse is the microsecond spike at the onset of the pulse [Fig. 3(a)] which is characteristic of flashlamp-pumped Ho:YAG lasers. The microsecond spike has a peak intensity that is ∼5× greater than that of the diode laser and thus a greater peak power. To determine whether the spike played a role in the stimulation efficacy of the Ho:YAG laser, the spike was removed from the pulse and experiments repeated with spikeless pulses (see Sec. 2.3.3).

**Fig. 7:**
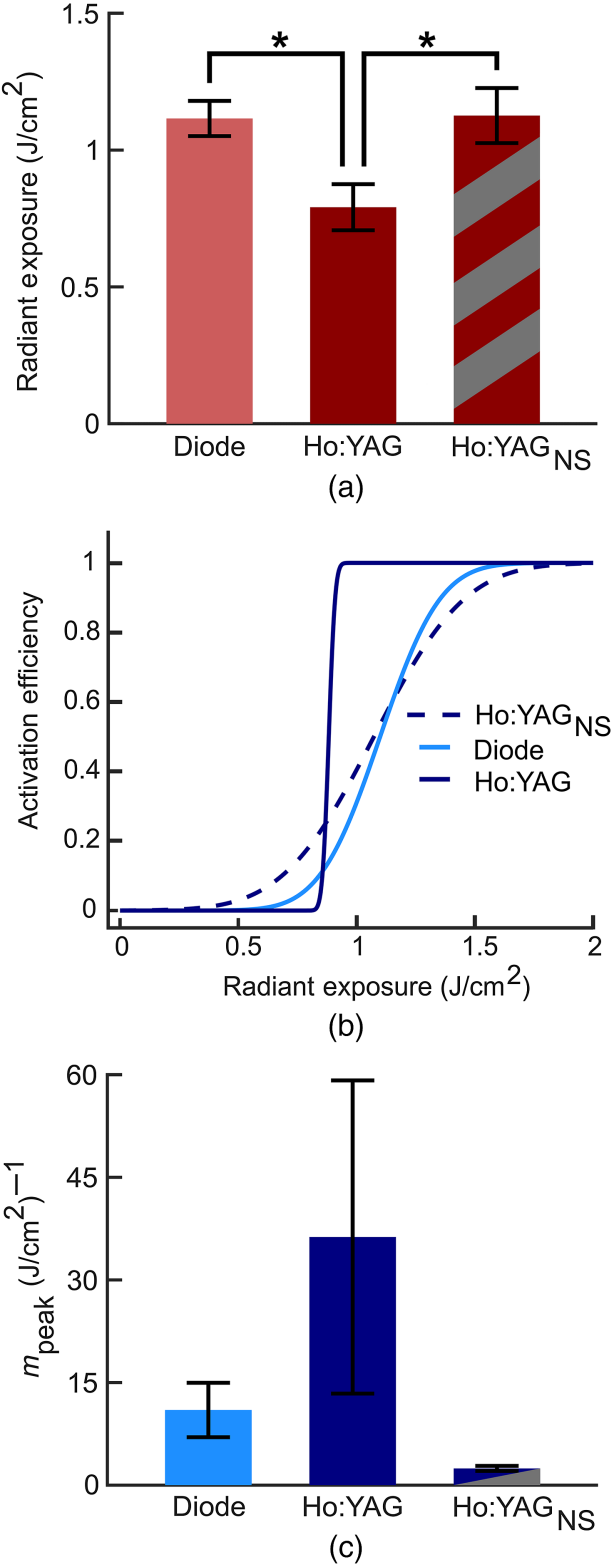
The effects of spikeless Ho:YAG pulse on stimulation efficacy. (a) Spikeless pulses produced a greater $H_{50}$ than the unaltered Ho:YAG pulse ($H_{50}\pm \mathrm{SEM}$, n=5) (b) Fitted CDFs to all data for the spikeless Ho:YAG, unaltered Ho:YAG, and diode lasers. (c) The transition rate ($m_{\text{peak}}$) of the spikeless Ho:YAG, unaltered Ho:YAG, and diode lasers ($m_{\text{peak}}\pm \mathrm{SEM}$, n=5). For all experiments: spot size=500  μm, $\lambda_{\text{Diode}}=1470\;\mathrm{nm}$, $\tau_{p}=350\;\mu s$. NS, spikeless.

The spikeless Ho:YAG pulse produced a significantly higher $H_{50}$ than the unaltered Ho:YAG pulse [p=0.025; Fig. 7(a)]. Moreover, the $H_{50}$ of the spikeless pulses did not differ compared to the $H_{50}$ of 1470 nm at the same 350  μs pulse width and same 500  μm spot size (p=1). The spikeless Ho:YAG pulse also yielded notable differences in activation probability. The $m_{\text{peak}}$ of the spikeless Ho:YAG pulses was remarkably less than that of the normal Ho:YAG pulses [Fig. 7(c)]. The $m_{\text{peak}}$ of the spikeless pulses was similar to the diode laser at an equal pulse width. The change in $m_{\text{peak}}$ of the spikeless Ho:YAG pulse to mirror that of the diode pulse is visually apparent in the CDFs fitted to each dataset [Fig. 7(b)].

### 3.5 Histological Damage Assessment

**Fig. 8:**
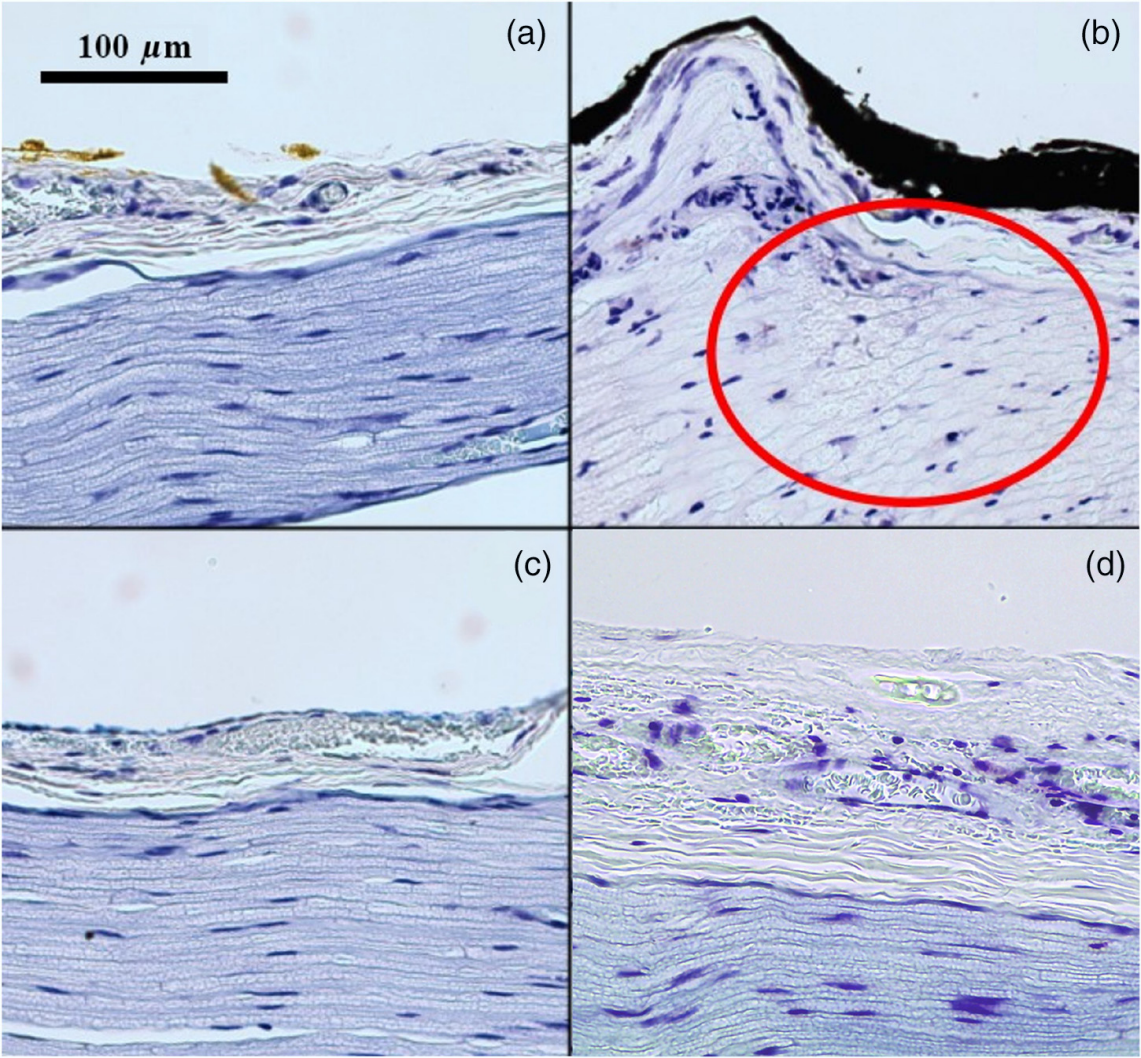
Representative irradiated nerve slices stained with toluidine blue. (a) Negative control, no stimulation. (b) Positive control: $6\;J/{\mathrm{cm}}^{2}$ at 1450 nm light. Red ellipse indicates region of myelin disruption. (c) $3.08\;J/{\mathrm{cm}}^{2}$ irradiation at 1450 nm. (d) $3.15\;J/{\mathrm{cm}}^{2}$ irradiation at 1875 nm.

Histological safety was evaluated by assessing myelin integrity using a toluidine blue stain. No signs of damage were observed in the negative control [Fig. 8(a)]. Clear evidence of charring and hyalinization were observed in the positive control at $∼6\;J/{\mathrm{cm}}^{2}$ [Fig. 8(b)]. Neither disruption of myelin sheath nor charring was observed for 1875 or 1450 nm even at three times the $H_{50}$ threshold [$3\;J/{\mathrm{cm}}^{2}$; Figs. 8(c) and 8(d)]. Results were consistent across all samples irradiated at one, two, and three times the $H_{50}$ radiant exposure. These results imply that INS with both 1875 and 1450 nm have a safety ratio of ∼3:1.

## 4 Discussion

Optical neuromodulation has had a profound impact on both clinical and basic research due to its spatial selectivity. In clinical applications where high spatial resolution is required including prostatectomies,^16^^,^^33^^,^^34^ rhizotomies,^29^ and skull base surgeries,^35^ optical methods could offer a superior alternative to electrical stimulation. With optogenetics, selective neural excitation requires targeting genetically altered neurons. This genetic specificity, however, presents translational difficulties in humans due to unknown immune reactivity and potential ethical constraints. INS circumvents these limitations by offering high spatial selectivity without the need for genetic modification or exogenous substrates. For these reasons, INS is a particularly well-suited method for clinical translation. Hence, in this study, we determined the optimal parameters for INS efficacy while confirming the identified parameters did not risk damage. To that end, the results of this study show shorter pulse durations improve activation probability and diode lasers exhibit a ∼3:1 safety ratio regardless of spot size.

Both the 1450 nm and 1875 nm diode lasers have a consistent $H_{50}$ of $∼1\;J/{\mathrm{cm}}^{2}$ across all considered spot sizes (Fig. 4). Thus, in broadening the spot size, the primary compromise is sacrificing the intrinsic spatial selectivity of INS. Since water serves as the primary chromophore in the SWIR responsible for the heat generation needed for INS, we initially expected 1450 nm, which has a higher water absorption coefficient than 1875 nm, to have a lower $H_{50}$. Moreover, scattering is considered negligible in the SWIR as absorption coefficients are considerably higher than the effective scattering.^18^ However, when the source term, S(z), given by

$$S(z)=\mu_{a}H_{0}e^{-\mu_{a}z},$$

which describes the absorbed energy density at a given depth in $J/{\mathrm{cm}}^{3}$ where $H_{0}$ is the initial radiant exposure and z is the depth of laser penetration in tissue, is compared for the two wavelengths along with the anatomy of the rat sciatic nerve,^69^ the deposited energy from each wavelength within the nerve fascicle itself is nearly equivalent (Fig. S3 in the Supplemental Materials). As the absorbed energy is the driving force underlying heat generation and thus INS, this comparison is of more consequence than the relative absorption coefficients. In addition, the endpoint used to determine successful stimulation is a CMAP which is a secondary and collective response from the population of excited axons. Thus, CMAPs are not dependent on the activation of a single axon at a specific depth but rather the activation of multiple axons over the entire irradiated volume. The similarity in $H_{50}$ of the three diode lasers used here is likely due to the fact that a similar amount of energy is deposited within the nerve fascicles themselves. The $H_{50}$ of the Ho:YAG laser, however, is lower than that of the diode lasers at $∼0.7\;J/{\mathrm{cm}}^{2}$, which is consistent for every spot size. For the 800 μm and 1000 μm spot sizes, this difference between the $H_{50}$ of the diodes and Ho:YAG lasers is statistically significant (p<0.02 for all comparisons). This difference, at first glance however, seems to contradict the initial observations of Wells et al.^18^

Wells et al. did not observe the same difference in the stimulation threshold between diode lasers and Ho:YAG lasers in the same *in vivo* rat sciatic nerve model.^18^ Rather the stimulation threshold was consistently between 0.4 and $0.5\;J/{\mathrm{cm}}^{2}$ for both lasers (see Fig. 2 in Ref. 18). The difference in reported values, however, is due to a difference in nomenclature and radiant exposure calculation. Here, the stimulation threshold refers to the $H_{50}$ or the radiant exposure at which 50% of the pulse will evoke a CMAP, whereas Wells et al. defined the stimulation threshold as the lowest radiant exposure to evoke a CMAP. Hence, the $H_{50}$ is expected to be greater than the stimulation threshold values reported by Wells et al. In adopting the stimulation threshold definition and spot size calculation of Wells et al., our data yield similar results with a stimulation threshold of 0.32  J/cm and 0.39  J/cm for the Ho:YAG laser and 1875 nm diode laser with 2 ms pulses, respectively. Moreover, whereas the spot sizes measured here were determined using the knife-edge technique, Wells et al. calculated their values based on the fiber diameter, the distance from the fiber to tissue, and the numerical aperture of the fiber. As the radiant exposure is inversely proportional to the square of the beam radius, the difference in calculated versus measured values also likely accounts for any discrepancies in the reported values. Nonetheless, seeing the evident difference between the $H_{50}$ of the diode lasers and the Ho:YAG observed here, the influence of pulse width on the efficacy of INS was then investigated.

While the pulse width of the Ho:YAG is fixed at 350  μs, the diode lasers have an adjustable pulse width. To create the necessary range of radiant exposures for the CDFs at each spot size in Fig. 4, the pulse widths of the 1875 nm diode laser were modulated. For the 500  μm, 800 μm, and 1000 μm spot sizes, the pulses were 2 ms, 3 ms, and 5 ms, respectively. Previously, Wells et al. also showed that the pulse width does not alter the stimulation threshold for INS with a constant spot size.^18^ Here, Fig. 4 similarly shows that even with changes in spot size and pulse width, the $H_{50}$ is unaffected for a given laser type (p=0.34). To confirm that the improved efficacy of the Ho:YAG is not due to its shorter pulse width, the $H_{50}$ of the Ho:YAG is compared to a 1470 nm diode laser capable of generating equal radiant exposures at a 350  μs pulse width. The data reaffirm that a pulse width of 350  μs versus 2 ms offers no advantage in terms of lowering the $H_{50}$ with the 1470 nm diode laser [Fig. 5(a)]. In Fig. 5(b), the $H_{50}$ for the Ho:YAG remains lower than the $H_{50}$ of the 1470 nm even with an equal pulse width, spot size, and radiant exposures. In addition to both lasers generating comparable optical energy for INS, 1470 nm light also has an almost equivalent water absorption coefficient to 2120 nm (Table 1). Consequently, this makes it unlikely that any observed differences are due to changes in optical absorption, penetration depth, or heat generation within the tissue. Taken together, the shorter pulse width of the Ho:YAG is not responsible for its lower $H_{50}$.

Though the $H_{50}$ across all lasers are unaffected by pulse width and spot size, the transition in activation probability (i.e., $m_{\text{peak}}$) increases as the pulse width of the diode lasers decreases (Fig. 6). Figure 6(a) depicts this observation graphically by plotting the CDF fitted to the combined data points from all trials of identical experimental conditions. As the pulse width decreases from 5 ms to 350  μs, the CDFs corresponding to the diode lasers transition to an activation probability of 1 more sharply over a smaller range of radiant exposures (i.e., have a steeper slope/$m_{\text{peak}}$). In other words, the response becomes more binary as the pulse width gets shorter, and a stimulation threshold is more readily identifiable. The rate of this transition is represented by the $m_{\text{peak}}$ of each CDF [Fig. 6(b)]. The smaller $m_{\text{peak}}$ of shorter pulse widths suggests that shorter pulse widths more consistently satisfy the conditions for the thermal gradient needed to generate an action potential. This observation complements the previous findings of Shapiro et al., who showed that for pulses ≤2  ms, the evoked inward current was shorter in duration and greater in amplitude than 10 ms pulses at 1889 nm.^22^ Moreover, the evoked currents for lower energy (1.4 mJ and 2.8 mJ) pulses ≤2  ms were greater despite the higher energy (7.3 mJ) of the 10 ms pulse. Inward currents resulting from a 10-ms pulse were substantially longer in duration and mirrored the square shape of the pulse, suggesting that pulse width plays a crucial role in the duration and amplitude of the evoked depolarizing currents. Because INS-induced inward currents are responsible for action potential initiation, the shorter duration and larger magnitude inward currents elicited from pulse widths ≤2  ms may explain why diode lasers more reliably excite CMAPs at shorter pulse widths and why the $m_{\text{peak}}$ is smaller for the 5 ms pulses examined here [as shown in Fig. 6(b)]. The reliability of shorter pulse durations could also have a thermodynamic explanation. The shorter pulse durations deposit infrared light within the tissue more quickly, allowing less time for thermal diffusion and potentially allowing a larger portion of the optical energy to directly generate the thermal gradient. However, even at an equal pulse width, the Ho:YAG consistently exhibited a much sharper transition in activation probability than the diode lasers.

The Ho:YAG generates an almost “all-or-none” response in which either every pulse will evoke a CMAP or none will. Figure 6(a) shows this feature of the Ho:YAG where the CDF resembles a step function. Further, the Ho:YAG has a greater $m_{\text{peak}}$ than the diode laser at an equal pulse width [Fig. 6(b)]. The sharper transitions of the Ho:YAG corresponds to more reliable stimulation (activation probability of 1) than the diode systems when only slightly above the $H_{50}$ and removes any ambiguity in achieving stimulation. This observation has clear clinical and experimental implications when using the Ho:YAG laser; INS is more likely to have an activation probability of 1 at a lower radiant exposure than the diode lasers. Though not on par with Ho:YAG laser, the higher power output of diode lasers centered near 1450 nm is a clear advantage over those centered near 1875 nm. Diode lasers at 1450 nm generally have a greater power output than 1875 nm diode lasers enabling them to produce the necessary radiant exposures needed at shorter pulse widths. Consequently, diode lasers centered near 1450 nm can achieve a higher $m_{\text{peak}}$ and sharper transitions in activation probability than 1875 nm diodes. Changing the pulse width of the 1450 nm diode laser, however, did not fully account for the larger $m_{\text{peak}}$ seen when using the Ho:YAG laser as compared the 1450 nm diode laser.

Figure 3(a) shows typical 350  μs pulse shapes from the 1470 nm diode and Ho:YAG lasers. Both pulses contain equal integrated areas and thus would deliver equal energies per pulse. The trace from the diode laser resembles a uniform square pulse mirroring the driving current. Conversely, the Ho:YAG pulse has an asymmetric shape with a ∼1  μs spike at the onset of the pulse. This pulse shape is a result of the Ho:YAG being pumped by a flashlamp as compared to a driving current. As the spike produces a higher peak power, its contribution to the stimulation efficacy of the Ho:YAG was investigated. Unfortunately, due to the rise-time of the diode, these lasers cannot generate similar pulse shapes. Instead, the effect of removing the spike in the Ho:YAG pulse was investigated. Experiments using spikeless Ho:YAG pulses resulted in a $H_{50}$ nearly equivalent to the 350 μs pulses of the 1470 nm diode laser [Fig. 7(a)]. Moreover, the $H_{50}$ from the spikeless pulse resulted in a significantly greater $H_{50}$ compared to the unaltered Ho:YAG pulses. The elimination of the microsecond spike also lowered the $m_{\text{peak}}$ of the Ho:YAG and once again produced results more similar to the diode laser. Consequently, both the spikeless Ho:YAG and diode laser pulses have a notably lower $m_{\text{peak}}$ than the unaltered Ho:YAG pulses [Fig. 7(c)]. Taken together, the results suggest that the microsecond spike at the onset of Ho:YAG pulses is largely responsible for the superior stimulation efficacy of the Ho:YAG laser compared to the diode lasers.

Based on the data, the lower $H_{50}$ and higher $m_{\text{peak}}$ of the Ho:YAG laser may be due to the rapid deposition of energy from the initial high intensity spike of the pulse. This finding aligns well with previous studies by Izzo et al.^70^^,^^71^ Using a 1937 nm diode laser, Izzo et al. demonstrated that the stimulation threshold to elicit a CAP in the gerbil cochlea decreases with decreasing pulse widths.^71^ Interestingly, they also noted a significant decrease in the stimulation threshold going from 300  μs to 100  μs pulses that gradually continued to decrease down to 5  μs pulse durations. In another study, Izzo et al. saw the same trend using a 1860 nm diode laser again in the gerbil cochlea this time noting that the peak power at the stimulation thresholds was constant from 100 to 1000  μs pulses but increased for pulse widths ≤35  μs.^70^ The Ho:YAG pulse, with its leading microsecond spike, may elicit a similar effect as observed by Izzo et al., creating a higher peak power and therefore lowering the stimulation threshold as seen at pulse durations <100  μs. Again, this effect could be the result of higher peak powers more consistently generating the thermal gradient needed for INS. Moreover, based on their data, Izzo et al. suggested that the thermal relaxation time for their optical target is in the range of ∼30 to 100  μs.^71^ Thus, with shorter pulse widths (≤30  μs) like the microsecond spike in the Ho:YAG pulse, the optical energy contained within the pulse is deposited within the nerve creating the required thermal rise well before the heat has time to dissipate (i.e., the pulse width is less than thermal relaxation time constant of the tissue). Given the controversy surrounding the mechanism of INS in the auditory system,^25^ it should be noted that Izzo et al. did not use a deafened animal model, and an opto-acoustic or thermal expansion model is consistent with the minimum power required for intracochlear stimulation, even though the pulse durations used do not meet conditions of stress confinement at this wavelength.^72^

These observations also complement the research done with photoabsorber-induced neurothermal stimulation (PAINTS).^73^^,^^74^ Similar to INS, PAINTS utilize exogenous chromophore absorption to achieve photothermal neural stimulation in order to provide more flexibility in targeting deeper neural structures. In cultured rat brain slices, Farah et al. demonstrated that the stimulation threshold for rat brain slice cultures decreases with pulse width and plateaus around 50  μs while using a 800 nm titanium-sapphire laser.^73^ Furthermore, pure thermal stimulation threshold curves failed to predict the results obtained from these short stimulation pulses suggesting an auxiliary nonthermal mechanism. Farah et al. hypothesize that the auxiliary mechanism may be photomechanical in nature as microsecond pulses are more likely to induce pressure wave transients than longer millisecond pulses. The data presented in this current study appear to lend credence to this hypothesis.

As stated previously, Shapiro et al. demonstrated that lower energy pulses ≤2  ms evoked inward currents shorter in duration and higher in amplitude than higher energy 10 ms pulses.^22^ Given the data presented here and assuming this trend holds, there is substantial evidence that the initial microsecond spike of the Ho:YAG pulse may be responsible for the lower $H_{50}$ and sharper transitions in the activation probability of the Ho:YAG laser stimulation. Moreover, if the spike is considered independently from the rest of the pulse, a microsecond pulse is much closer to the stress confinement regime than the other pulse durations considered here. Though no empirical evidence is offered here, weak photomechanical effects may exist during Ho:YAG stimulation as photomechanical and photothermal effects are not easily decoupled. Therefore, Ho:YAG stimulation could possibly initiate action potentials through the same universal INS mechanism proposed by Plaksin, Shoham, and Kimmel, via a partially photomechanically driven rather than a solely photothermally driven capacitance change.^23^ The reader should note, however, that data presented here and that by Shapiro et al. use substantially different experimental setups.

In translating INS for clinical applications, the data from the standard and spikeless Ho:YAG experiments suggest that brief low-energy pulses with durations on the order of a microsecond may yield a more reliable approach for INS. The microsecond spike only contains 8% to 11% of the total pulse energy and yet has a profound effect on the stimulation efficacy. If INS only requires short, low-energy pulses, then applying this technique becomes highly advantageous for clinical applications as lower energies further reduce the risk of laser-induced damage. At the time of writing, however, the authors are unaware of any cost-effective laser source capable of producing the combination of low energy and short pulses at appropriate wavelengths. Though not investigated as part of this study, using a collimated beam also has practical, clinical implications. Radiant exposure is inversely proportional to the square of the beam radius incident on the tissue. With a bare optical fiber, the incident spot is dependent on the distance of the fiber output to the tissue. Thus, in handheld probes, slight changes in the distance of the fiber from tissue, as is common during clinical procedures, can drastically change the radiant exposure. A collimated beam provides a consistent spot size over a wide range of distances keeping the magnitude of the radiant exposure constant over multiple fiber placements or adjustments.

Lastly, we demonstrate the acute histological safety of 1450 nm light for INS for the first time (Fig. 8). Histology slides were examined using a binary grading scheme similar to previous studies where signs of damage included granular disruption and vacuolization of myelin sheaths of nerve fibers, disruption of axons, charring, and ablation crater formation.^57^ At radiant exposures three times their $H_{50}$ ($∼3\;J/{\mathrm{cm}}^{2}$), the 1450 nm and 1875 nm diode lasers show no signs of myelin disruption or charring [Figs. 8(c) and 8(d)]. Myelin disruption was observed at radiation exposures $>3\;J/{\mathrm{cm}}^{2}$. Thus, both diode lasers behave comparably with a shared safety ratio of ∼3:1. The safety ratio identified here is similar to the acute safety ratio observed with the Ho:YAG laser, which has been thoroughly characterized in previous studies.^57^ This validates the use of diode lasers at 1450 nm and 1875 nm for INS despite their higher $H_{50}$ and lower $m_{\text{peak}}$ values as compared to the more effective Ho:YAG laser.

Taken together, these findings confirm that INS can effectively and safely excite neural activity in the rat sciatic nerve. The data corroborate previous conclusions concerning the effects of pulse duration and stimulation threshold while also showing that 1450 nm laser diodes can serve as a more useful source for INS than the 1875 nm diode lasers.

Regardless of the advantages of the Ho:YAG laser, the SWIR diode laser remains an effective, low-cost, and user-friendly means of INS. The results presented here provide valuable insight into maximizing the efficacy of diode lasers for INS. While the $H_{50}$ of the diode lasers is constant regardless of pulse width, shortening the duration of the pulse width leads to more reliable stimulation due to a higher transition rate in activation probability. INS at shorter pulse widths is especially achievable when using laser diodes centered near 1450 nm as these diodes can generate sufficient radiant exposures at shorter pulse widths than 1875 nm diodes. The spot size plays little to no role in the efficacy of INS but comes at the cost of spatial selectivity. For the first time, histological evidence that 1450 nm and 1875 nm exhibit similar damage thresholds and safety ratios is presented. The diode lasers have a safety ratio of 3:1 compared to the 2:1 safety ratio of the Ho:YAG.^57^ Since we have shown that 1450 nm and 1875 nm diode lasers are practically equivalent laser sources for INS, the wider commercial availability of 1450 nm diode and its higher power output can be leveraged to improve the accessibility and versatility of INS in both experimental and clinical settings.

## 5 Conclusion

We show that the 1450 nm diode lasers represent an effective and safe laser source for INS application on par with the more commonly used 1875 nm diode lasers. The Ho:YAG laser was shown to have greater stimulation efficacy than diode lasers primarily due to the initial spike at the onset of its pulse. Though the Ho:YAG laser outperformed the laser diode systems, diode lasers remain a cost-effective and reliable laser source for INS. These results also provide easy steps one can take to optimize the performance of diode lasers for INS. Looking forward, a compact, true pulsed laser would be the ideal laser system for clinical INS. The 1450 nm diode laser systems, however, provide an optimal compromise to explore INS strategies. Together, our results offer insight into further avenues for improving INS and its laser sources as a tool for label-free neural modulation.
