# Supplementary material for: Identifying optimal parameters for infrared neural stimulation in the peripheral nervous system
Source: Neurophotonics. 2021 Mar 31;8(1):015012. doi: 10.1117/1.NPh.8.1.015012 (PMC8010905; doi:10.1117/1.NPh.8.1.015012)
Supplement: Supplementary file 1 [file NPh_008_015012_SD001.pdf]

Supplementary Material

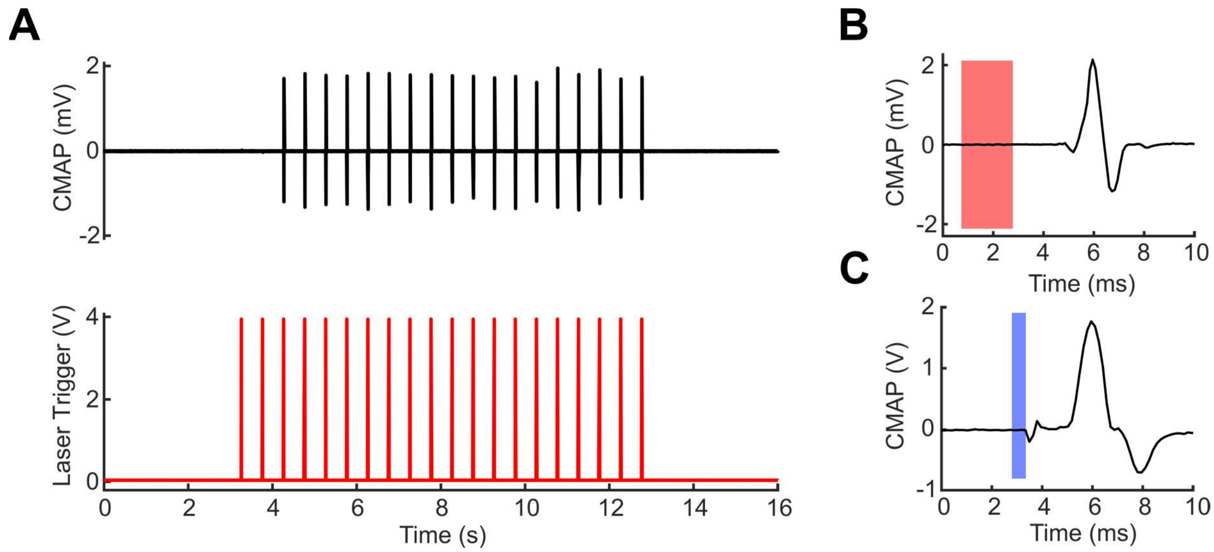

Figure S1. Representative CMAP traces. **(A)** Example of INS pulse train and corresponding evoked-CMAPs ($Spot\ Size = 800\ \mu m; \lambda = 1875\ nm;\ \tau_p = 3\ ms, H = 1.36\ J/cm^2$). **(B)** Representative single INS-evoked CMAP. Note absence of ES artifact. Red bar indicates duration of INS. ($Spot\ Size = 1000\ \mu m; \lambda = 1450\ nm;\ \tau_p = 2\ ms, H = 1.40\ J/cm^2$). **(C)** Representative trace from ES-evoked CMAP. Note presence of ES artifact. Blue bar indicates duration of ES. ($\tau_p = 500\ \mu s;\ V = 0.3\ V$).

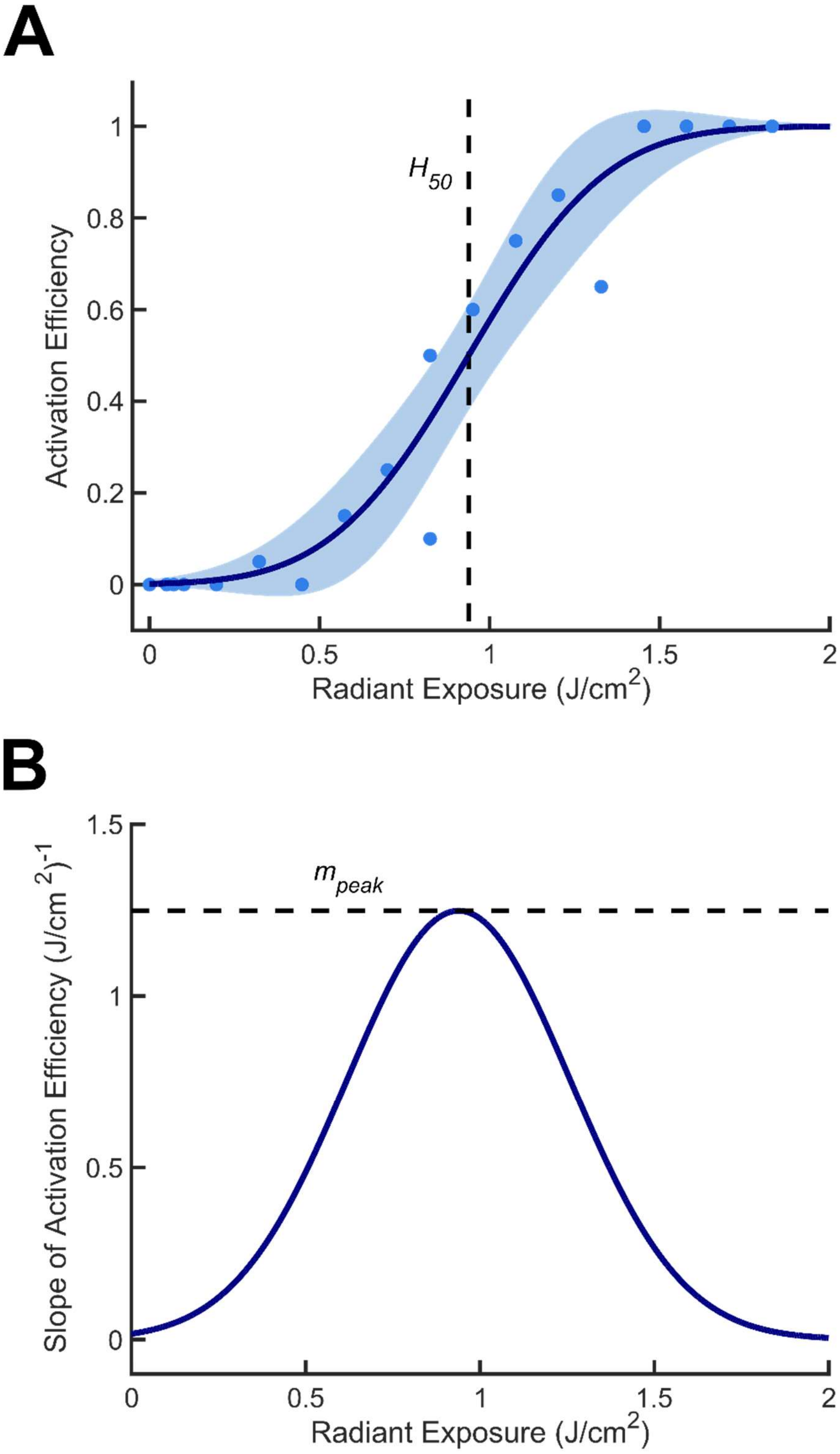

Figure S2. Graphic example of **(A)** H_{50} and **(B)** m_{peak} calculations. Shaded area in (A) is the standard deviation from the CDF fitting.

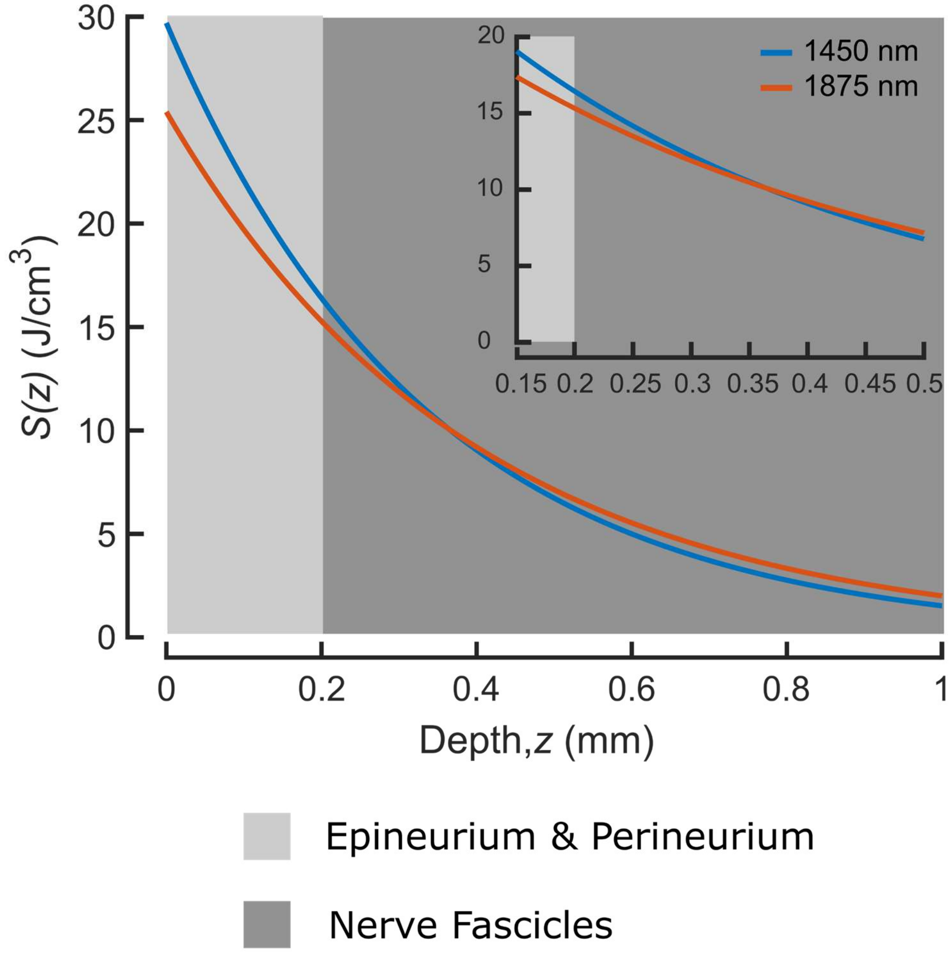

Figure S3. Source term $S(z)$ for 1450 nm and 1875 nm in rat sciatic nerve. Thickness of nerve layers were taken from Ref [21] and absorption coefficients from Ref [60].
